# The Record at the North

**Published:** 1878-06-20

**Authors:** 


### THE RECORD AT THE NORTH.

We are gratified at the increasing popularity of our journal, and the extension of its list among the busy practitioners in all sections of the Union. During the past year we had additions occasionally coming in from the extreme Northern States, giving evidence of the subsidence of those bitter sectional prejudices which in years past have prevailed between the sections. Perhaps there was less of this bitterness in our profession than among others. We give the following extract from a letter recently received from a medical man in Corning, N. Y. The name of the writer we do not take the liberty to publish, though it would honor both his head and heart, as we have not asked his permission to do so. We commend its sentiments to others of our profession:

"I am one of those who think that by every means possible the two sections of our common country should get closer together, and know each other better. I have observed that our profession have led all the others in the early restoration of amicable relations between the North and South, and none of us have been any the worse for it. I served nearly four years as a surgeon in the Union army during the late war, and made the acquaintance of many surgeons, officers and men from the South, and ever found them agreeable gentlemen, brave to a fault and patient to endure. My recollections of those trying times are not embittered by the reflection that I ever offered an indignity to a captured man, but sweetened by the consciousness that I treated every man in my care as best I could, making no distinction between Union and Confederate soldiers, our common humanity and professional demands making it obligatory that the golden rule be our standard in administering to those helpless and friendless."

